# Transoral laser microsurgery for the treatment of oropharyngeal cancer: the Dalhousie University experience

**DOI:** 10.1186/s40463-015-0093-3

**Published:** 2015-09-30

**Authors:** J.C. Melong, M. H. Rigby, M. Bullock, R. D. Hart, J. Trites, S. M. Taylor

**Affiliations:** Division of Otolaryngology - Head and Neck Surgery, Queen Elizabeth II Health Sciences Centre and Dalhousie University, Halifax, NS Canada; Division of Anatomical Pathology, Queen Elizabeth II Health Sciences Centre and Dalhousie University, Halifax, NS Canada

**Keywords:** Transoral laser microsurgery, Minimally invasive surgery, Oropharyngeal carcinoma, Head and neck surgery, Head and neck cancer

## Abstract

**Objective:**

The optimal treatment strategy for oropharyngeal squamous cell carcinoma is highly debated. However, growing evidence supports the use of minimally invasive techniques, such as transoral laser microsurgery (TLM), as a first-line treatment modality for these carcinomas. The purpose of our study was to assess the efficacy and safety of TLM for the treatment of primary and recurrent oropharyngeal carcinomas.

**Methods:**

All patients with oropharyngeal carcinoma undergoing TLM at the QEII Health Sciences Centre in Halifax, Nova Scotia were identified within a prospective database monitoring TLM outcomes. Kaplan-Meier survival analysis was used to evaluate the following end points at 36 months: local control (LC), disease-specific survival (DSS), and disease-free survival (DFS). Safety endpoints included complications following surgery and long term morbidity related to TLM.

**Results:**

Between 2003 and 2014, 39 patients with oropharyngeal carcinoma underwent TLM resection. Twenty-eight (72 %) patients had primary carcinoma, nine (23 %) were radiation/chemoradiation (RT/CRT) failures, and two (5 %) had second primaries following previous RT/CRT. Three patients had stage I disease, 8 stage II, 5 stage III, and 23 stage IV disease. HPV status was available for 26 patients, of which 23 (88 %) had HPV positive disease. Kaplan-Meier estimates of 36-month LC, DSS, and DFS for primary oropharyngeal carcinomas were 85.5 % (SE 10.6 %), 85.7 % (SE 13.2 %) and 77.7 % (SE 12.5 %) respectively. Thirty-six-month outcomes for RT/CRT failures were 66.76 % (SE 15.7 %) for LC and 55.6 % (SE 16.6 %) for DSS and DFS. Three patients developed complications following surgery.

**Conclusions:**

Observed 36-month efficacy and safety outcomes support the use of TLM for the treatment of primary and recurrent oropharyngeal carcinoma.

## Introduction

The incidence of oropharyngeal squamous cell carcinoma (OSCC) has been increasing, largely because of increasing rates of Human Papilloma Virus (HPV) infection [1–4]. This is an important trend clinically as HPV positive OSCC is associated with a better prognosis and treatment outcome [5, 6]. In the past, the management of OSCC has largely been guided by treatments that minimized functional morbidities. Radiation/chemoradiation (RT/CRT) was often preferred because historically, surgical intervention involved open en bloc resection of the tumour with free flap reconstruction, resulting in significant compromise of surrounding structures and ultimately function. However, RT/CRT has been associated with significant acute and long term toxicities and decreased quality of life [7–10].

More recently, transoral laser microsurgery (TLM) has emerged as a minimally invasive, endoscopic surgical technique for the management of oropharyngeal and laryngeal carcinomas. Initially used for carefully selected early stage head and neck cancers, experience with TLM has expanded its use to include select advanced lesions [11, 12]. Contraindications to TLM include inadequate access to the primary site (e.g. trismus, large tongue base, prominent dentition, etc.), large vessel proximity or involvement (e.g. tumour adjacent to the carotid bulb or internal carotid, deep bilateral base of tongue invasion increasing the risk of damage to both lingual arteries, etc.), and oncologic contraindications including T4b cancers, unresectable neck disease or multiple distant metastases [13, 14]. Despite these limitations, TLM offers potential advantages over open surgery including shorter recovery time, fewer complications and better functional outcomes [15–17]. Similarly, preliminary studies have demonstrated TLM to have improved functional outcomes compared to RT/CRT, particularly with respect to swallowing function [18, 19]. Surgical intervention also offers the advantage of pathologic characterization of the tumour, helping to guide further management and treatment, an advantage that is not readily available with RT/CRT.

The purpose of this study was to evaluate the oncologic measures of TLM for OSCC at our center, adding to the growing evidence of support for the use of TLM as a first line treatment modality for these carcinomas.

## Methods

This was a prospective, cohort study based on a database monitoring all malignancies treated with TLM at the QEII Health Sciences Centre in Halifax, Nova Scotia. The collection of information within the database was approved by our institutional research ethics board. The database was created in 2005 and has been prospectively maintained since that time. Information prior to 2005 was collected retrospectively at the time of the creation of the database. Details regarding data collection and the information contained within the database have been described previously [20, 21].

Between January 2002 and December 2014, approximately 300 different patients with suspected upper aerodigestive tract malignancies were treated with TLM by the senior author at our centre. All cases of primary or recurrent OSCC treated with TLM were included in the current study. Exclusion criteria included malignancies in all other sites of the upper aerodigestive tract treated with TLM.

## TLM, neck dissection and adjuvant therapy

All patients in the cohort underwent TLM resection. At the time of resection, a FK Retractor and/or Bouchayer laryngoscope was used to obtain adequate tumour exposure. Once adequate exposure was obtained, tumours were excised with a CO2 laser using a tumour-splitting approach. Margin status was determined at the time of surgery through frozen section analysis. Positive frozen margins were subsequently resected until negative margins were obtained. Following surgery, tissue submitted for intraoperative margin analysis was resubmitted for routine processing. The main resection specimen was also submitted in toto for pathological characterization of the tumor.

Neck dissections were done concurrently at the time of TLM resection. In keeping with current guidelines, all patients were considered for concurrent neck dissections unless they had previous radiation failure or had no evidence of clinical or radiologic neck disease [22]. The exception were patients who had previous radiation failure, but stopped radiation early because of side-effects or presented with new or recurrent neck disease for which a neck dissection was not previously done. Patients with primary tonsil carcinoma underwent ipsilateral neck dissections while patients with base of tongue carcinomas underwent bilateral neck dissections. External carotid branches were ligated at the time of the neck dissection, including ligation of lingual and facial arteries.

Adjuvant therapy was offered to patients who met one or more of the following criteria, unless there was a contraindication to therapy: advanced disease, multiple nodal involvement, extracapsular extension from involved lymph nodes, perineural invasion, or positive margins following TLM resection. This is in keeping with current evidence that demonstrates improved survival with adjuvant therapy in these select patients [22–25].

## Analysis

A descriptive analysis of demographics, morbidities, and outcomes was performed. HPV status was determined by p16 immunohistochemistry. HPV positive disease was defined by strong, diffuse nuclear and cytoplasmic positivity (>70 %) of tumor cells for the p16 marker. Smoking status was self-reported by patients and categorized into current, past or non-smoking status at the time of surgery. Margin status was determined by reviewing individual pathology reports for all patients in the cohort. Close margins were defined as any margin less than 5 mm.

All statistical testing was performed using an intention-to-treat analysis. Kaplan-Meier 36-month survival analysis was performed for the following end points: local control (LC), disease-free survival (DFS), and disease-specific survival (DSS). Local control was defined as local recurrence-free survival obtained with one TLM resection or with subsequent reresection(s) for positive or close margins. Disease-free survival was defined as no local or regional recurrence or presence of a new second primary oropharyngeal tumour. Cancers were defined as a second primary tumour if they occurred more than 5 years after the last received treatment or if they occurred on the contralateral side to a previously treated unilateral tumour that did not cross the midline.

## Results

Between 2002 and 2014, 39 patients (31 males and 8 females) with oropharyngeal carcinoma underwent TLM resection (Table 1). The mean age of the cohort at the time of diagnosis was 59.6 years (range 32–80). Twenty-eight (72 %) patients had primary carcinoma, 9 (23 %) were RT/CRT failures, and 2 (5 %) had second primaries following previous RT/CRT. Three patients had stage I disease, 8 stage II, 5 stage III, and 23 stage IV. HPV status was available for 26 patients, of which 23 (88 %) had HPV positive disease. The mean time of follow-up was 23 months. Among patients with primary OSCC, 21 patients received adjuvant therapy; 14 received radiation therapy and seven received chemoradiation. Twenty-nine (75 %) patients underwent concurrent neck dissections at the time of TLM resection. The average length of hospital stay was 3.8 days (range 2–10 days).

**Table 1:** Patient characteristics (*n* = 39)

Characteristic	Number of patients (%)
Age, y	
Mean (Range)	59.6 (32–80)
Sex	
Male	31 (79.5 %)
Female	8 (20.5 %)
Tumour subsite	
Base of tongue	20 (51 %)
Tonsil	19 (49 %)
Stage	
I	3 (7.5 %)
II	8 (20.5 %)
III	5 (13 %)
IV	23 (59 %)
T Stage	
T1	9 (23 %)
T2	23 (59 %)
T3	7 (18 %)
T4	0 (0 %)
N stage	
N0	13 (33 %)
N1	3 (8 %)
N2a	5 (13 %)
N2b	13 (33 %)
N2c	2 (5 %)
N3	3 (8 %)
HPV status (*n* = 26)	
Positive	23 (88.5 %)
Negative	3 (11.5 %)
Smoking status (*n* = 37)	
Current smoker	6 (16 %)
Past smoker	20 (54 %)
Lifetime nonsmoker	11 (30 %)

Three patients developed complications following TLM resection. One patient experienced significant postoperative bleeding requiring a blood transfusion 14 days postoperatively. The patient was taken to the OR for exploration, but no active site of bleeding could be identified. The bleeding resolved spontaneously and was later determined to be caused from a longstanding gastric ulcer. Two patients developed cardiovascular complications. One patient experienced a myocardial infarction following TLM reresection of a positive margin and another patient developed a pulmonary embolism 3 days postoperatively. All patients recovered from their complications.

Two patients required a gastrostomy tube (G-tube) postoperatively following initial TLM resection. One patient required a temporary G-tube (<2 months) following TLM resection for swallowing difficulties. The other patient required a long-term G-tube following postoperative CRT for a close margin. One and two year G-tube rates following initial TLM resection were 3 and 0 %, respectively. Three patients required G-tubes following salvage therapy for recurrences. One patient required a temporary G-tube (<6 months) following salvage radiation therapy for a local recurrence. Another patient, who underwent a radical neck dissection following recurrence of a neck mass, subsequently developed a hematoma postoperatively and required a tracheostomy and G-tube for breathing and swallowing difficulties. Finally, one patient who ultimately underwent a total laryngopharyngectomy for new primary disease, required a G-tube postoperatively after the development of a tracheoesophageal fistula. Of note, one patient developed mild velopharyngeal insufficiency postoperatively that improved overtime without intervention and another patient, who was a previous CRT failure with a preoperative G-tube, was able to have their G-tube successfully removed following TLM resection.

Five patients had temporary tracheostomies following TLM. Initially, this was done for all TLM resections for anticipated postoperative swelling. This practice was stopped after it was seen that most patients had minimal swelling postoperatively. All five patients had their tracheostomies removed prior to discharge. One and two year tracheostomy rates were 0 %.

**Fig. 1:**
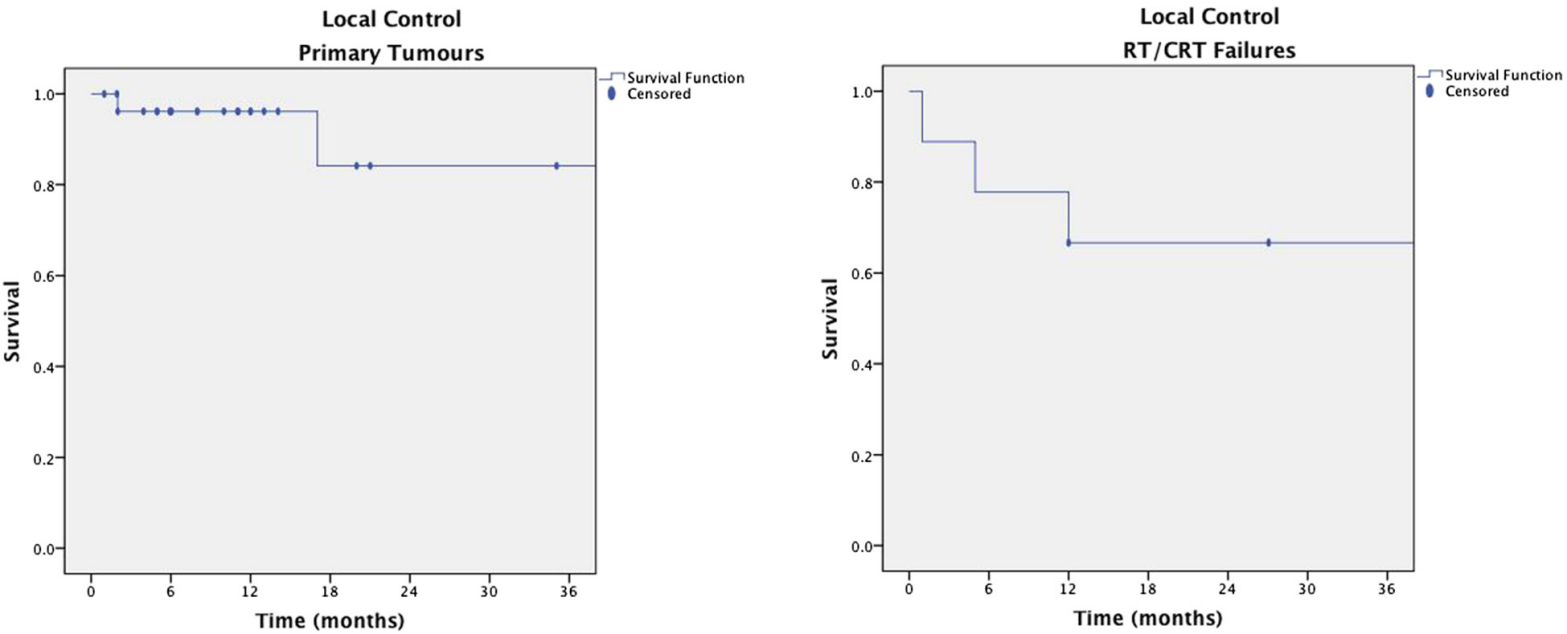
Kaplan-Meier estimates of 36-month local control

There were eight cases of recurrence following TLM, including five local recurrences (two of which also had regional recurrence), two regional recurrences, and one case of metastasis. There was also one case of a new primary in a patient with a right tonsil OSCC who went on to develop a left piriform sinus OSCC following TLM. Recurrence was more common among RT/CRT failures compared to patients with new primary oropharyngeal carcinoma. Kaplan-Meier estimates of 36-month LC for new primary oropharyngeal carcinoma was 85.5 % (SE 10.6 %) compared to 66.76 % (SE 15.7 %) for RT/CRT failures (Fig. 1).

**Fig. 2:**
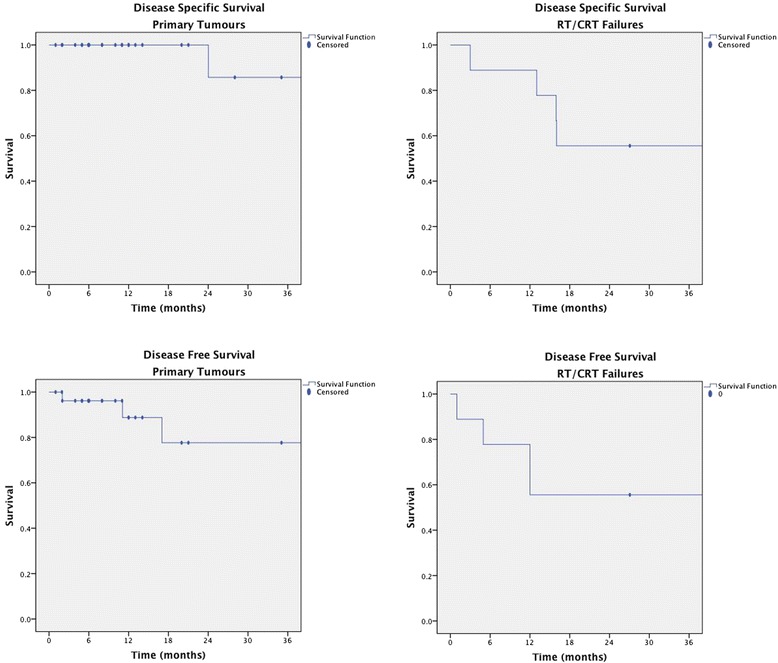
Kaplan-Meier estimates of 36-month disease specific survival and disease free survival

During follow-up, seven patients died from their disease. All four RT/CRT failures who developed recurrence died from their disease. Two of four patients with primary oropharyngeal carcinoma who developed recurrence died from their disease. One patient, who received TLM for a second primary following previous CRT, developed a new primary following TLM and despite undergoing a total laryngopharyngectomy, ultimately developed metastatic disease and died from their disease. Kaplan-Meier estimates of 36-month DSS and DFS for primary oropharyngeal carcinoma were 85.7 % (SE 13.2 %) and 77.7 % (SE 12.5 %) compared to 55.6 % (SE 16.6 %) and 55.6 % (SE 16.6 %) for RT/CRT failures (Fig. 2).

Adjuvant therapy was administered to 21 of 28 patients with new primary OSCC (Table 2). Fourteen patients received radiation therapy and seven received chemoradiation. The remaining seven patients received no postoperative therapy. Among patients who did not receive postoperative therapy, three developed recurrence. All three patients had stage III/IV oropharyngeal carcinoma and of these, two patients refused adjuvant therapy for personal reasons and one was not a candidate because of comorbid health conditions. No patients with stage I/II disease treated with TLM monotherapy developed recurrence. Among patients who received adjuvant therapy, one patient developed metastatic disease.

**Table 2:** Adjuvant therapy for new primary OSCC following TLM resection (*n* = 28)

Adjunctive therapy	Number	Recurrence	New primary	Recurrence type	Laryngectomy	Death due to disease
None	7	3	0	2 Local	0	1
				1 Regional		
Radiation	14	0	0	0	0	0
Chemoradiation	7	1	0	1 Metastatic	0	1

Salvage therapy was carried out in two of four cases of recurrence among patients with primary oropharyngeal carcinoma. One case of local recurrence was salvaged with radiation therapy and the patient currently remains disease free. The other case of regional recurrence was salvaged with a selective neck dissection, but unfortunately the patient went on to develop metastatic disease and died from their disease. Among the other two cases of recurrence, one patient developed metastatic disease for which further therapy was not indicated and the other patient refused further treatment after local recurrence. As previously mentioned, a total laryngopharyngectomy was carried out in a patient who developed a new primary following TLM, but the patient ultimately developed metastatic disease and died from their disease. No previous RT/CRT failures who developed recurrence received salvage therapy.

Four patients (10 %) had positive margins and one patient (3 %) had a close margin at the primary site following initial TLM resection (Table 3). Four patients, including the patient who had a close margin, had primary OSCCs and one patient was a previous RT failure. The previous RT failure subsequently underwent TLM reresection, but ultimately developed locoregional recurrence and died from their disease. Among the four patients with primary OSCCs, three received postoperative chemoradiation and one received postoperative radiation. Two of the three patients who received postoperative chemoradiation stopped their chemotherapy early because of side-effects. Of the patients who received adjuvant therapy, one developed metastatic disease and died from their disease. The other three patients remain disease free.

**Table 3:** Intraoperative disease control

	Margins (−)	Margins (+)
Total	34	5
Local recurrence	3	0
Locoregional	1	1
Regional	2	0
Metastatic	0	1
New primary	1	0

Twenty three patients had HPV positive disease and three patients had HPV negative disease. HPV status could not be determined in 13 patients. Among HPV positive patients, two developed recurrence. One patient developed a local recurrence after initially refusing adjuvant radiation therapy and is currently awaiting further management. The other patient developed metastatic disease and died from their disease. Among the three patients with HPV negative disease, two developed recurrence. One patient developed recurrent regional disease and died from their disease. The other patient developed local recurrence and after receiving salvage radiation therapy, remains disease free.

## Discussion

Increasing evidence supports the use of transoral surgery as an effective, minimally invasive strategy for the treatment of OSCC. Currently, two transoral treatment modalities exist, TLM and transoral robotic surgery (TORS). Both transoral approaches have demonstrated excellent local control and overall survival for the treatment of primary OSCC, while minimizing functional compromise [11, 26–28]. Recent studies have also demonstrated TLM and TORS to be effective as salvage therapy for recurrences in previous RT/CRT failures [29, 30]. This current study adds to the growing body of evidence, demonstrating TLM to have high rates of local control and overall survival for both primary and recurrent oropharyngeal cancers with excellent functional outcomes.

Eight patients in the cohort developed recurrence. This relatively high rate of recurrence can be expected based on the increased risk of recurrence among RT/CRT failures, which has been previously demonstrated to have a worse prognosis [31, 32]. Among the nine RT/CRT failures in the study, four developed recurrence. Despite these findings, TLM still demonstrated good local control and overall survival for RT/CRT failures when compared to other available salvage treatment modalities [31, 32].

Among patients with primary OSCC, recurrence was higher in patients who did not receive adjuvant therapy. This can be partly explained by the high rate of recurrence among patients with advanced disease who refused adjuvant therapy. In the current cohort, all three patients with advanced primary disease who did not receive adjuvant therapy following TLM, either because they refused or were not a candidate for RT/CRT, developed recurrence. There was no recurrence in patients with stage I/II primary disease who received TLM monotherapy. This is consistent with previous studies that have demonstrated TLM to be effective as a single treatment modality for early disease [33]. Although it is difficult to draw conclusions from small numbers, this study provides further evidence for the benefit of TLM as a single treatment modality for early disease and the potential benefit of postoperative adjuvant therapy in select patients with advanced disease.

In the current cohort, 4 patients had positive margins (10 %) and one patient (3 %) had a close margin at the primary site following initial TLM resection. This is comparable to other studies, which have demonstrated positive margin rates to be between 15 and 30 % [34]. Similar to the findings in other studies, patients with positive margins in the current cohort tended to have bulkier tumours (T2 or T3) and node positive disease [34]. One of the major advantages of TLM over other treatment modalities is the ability to perform reresection in the presence of positive margins. This is particularly beneficial for patients with previous RT/CRT failure, as treatment modalities for OSCC recurrence is limited. In the present study, only one patient with a positive margin underwent reresection. Unfortunately, the patient subsequently developed locoregional disease and died from their disease. The other three patients who had positive margins and one patient who had a close margin following initial TLM resection would also generally be considered for reresection. However, all four patients had advanced diseased and required postoperative adjuvant therapy. As pathology reports at our centre generally take several weeks to obtain, it was decided that RT/CRT should be initiated before final pathology results were obtained. This proved to be an effective approach as three of the four patients with positive/close margins who received adjuvant therapy currently remain disease free. However, in early disease TLM reresection alone could be considered, helping to avoid the acute and long term complications related to RT/CRT.

Previous studies have demonstrated HPV positive OSCCs to have a better prognosis and treatment outcome compared to HPV negative disease [5, 6]. This trend was reflected in our study where only 9 % (2/23) of patients with HPV positive disease developed a recurrence compared to 66 % (2/3) of patients with HPV negative disease. Unfortunately, status was unknown for 13 patients as p16 immunohistochemistry was only introduced and done routinely at our centre for OSCCs since 2009. Obviously with such a small number of HPV negative patients in our study, it is difficult to draw meaningful conclusions. However, the study does demonstrate TLM to be highly effective for the management of HPV positive disease.

One of the limitations of our study is the relatively limited period of follow-up for a large percentage of the cohort. Although the cohort included all patients undergoing TLM between 2002 and 2014, more than half of the patients underwent initial TLM resection from 2012 onward. As a result, statistical analysis was only adequately powered for follow-up at 3 years. Despite this limitation, the current study provides compelling evidence for the efficacy and safety of TLM for primary and recurrent OSCCs.

## Conclusion

The optimal treatment strategy for oropharyngeal carcinoma is highly debated. However, growing evidence supports the use of TLM as a safe and effective first-line treatment modality for OSCCs. This study provides further evidence for the use of TLM as a first-line treatment modality for primary and recurrent OSCCs, demonstrating excellent 3-year survival and functional outcomes.

## Abbreviations

OSCC: Oropharyngeal squamous cell carcinoma
HPV: Human Papilloma Virus
RT/CRT: Radiation/chemoradiation
TLM: Transoral laser microsurgery
TORS: Transoral robotic surgery
LC: Local control
DFS: Disease-free survival
DSS: Disease-specific survival
