# Erratum to: Transoral laser microsurgery for the treatment of oropharyngeal cancer: the Dalhousie University experience

**DOI:** 10.1186/s40463-015-0095-1

**Published:** 2015-10-21

**Authors:** Jonathan C. Melong, Matthew H. Rigby, Martin Bullock, Robert D. Hart, Jonathan R.B. Trites, S. Mark Taylor

**Affiliations:** Division of Otolaryngology - Head and Neck Surgery, Queen Elizabeth II Health Sciences Centre and Dalhousie University, Halifax, NS Canada; Division of Anatomical Pathology, Queen Elizabeth II Health Sciences Centre and Dalhousie University, Halifax, NS Canada

Unfortunately, the original version of this article [1] contained an error. The names of the authors were included incorrectly and the following sentence was missing from the author’s contribution section: “SMT was the research supervisor and assisted with primary data acquisition, analysis and interpretation, and assisted with preparation and revision of the manuscript”.

The names and the author’s contribution section have been updated in the original article and are also correctly included in full in this erratum.
